# Diagnostic Value of Urinary Steroid Profiling in the Evaluation of Adrenal Tumors

**DOI:** 10.1007/s12672-015-0224-3

**Published:** 2015-05-19

**Authors:** T. M. A. Kerkhofs, M. N. Kerstens, I. P. Kema, T. P. Willems, H. R. Haak

**Affiliations:** 1 Department of Internal Medicine, Máxima Medical Center, Ds. Th. Fliednerstraat 1, 5631 BM Eindhoven, The Netherlands; 2 Department of Endocrinology, University Medical Center Groningen, University of Groningen, Groningen, The Netherlands; 3 Department of Laboratory Medicine, University Medical Center Groningen, University of Groningen, Groningen, The Netherlands; 4 Department of Radiology, University Medical Center Groningen, University of Groningen, Groningen, The Netherlands; 5 Department of Health Services Research and CAPHRI School for Public Health and Primary Care, Maastricht University, Maastricht, The Netherlands; 6 Department of Internal Medicine, Division of General Internal Medicine, Maastricht University Medical Centre+, Maastricht, The Netherlands

**Keywords:** Primary Aldosteronism, Adrenal Tumor, Adrenal Mass, Adrenocortical Carcinoma, Adrenal Incidentaloma

## Abstract

Radiological examination may unexpectedly reveal an adrenal mass. Current algorithms for differentiating between benign and malignant lesions mainly rely on size and densitometry on unenhanced CT, which have limited specificity. We examined the diagnostic value of urinary steroid profiling by gas chromatography/mass-spectrometry (GC/MS) in differentiating between benign and malignant adrenal tumors. A retrospective study in two referral centers for patients with adrenal disease was performed. All urinary steroid profiles ordered for evaluation of an adrenal tumor between January 2000 and November 2011 were examined. Patients were diagnosed with adrenal cortical carcinoma (ACC), adrenal cortical adenoma (ACA), or other adrenal mass. Results of hormonal measurements, imaging studies, pathology reports, and clinical outcome were retrieved from medical records. The diagnostic value of individual urinary steroid metabolites was determined by receiver operating characteristics analysis. Cut-off values were compared to reference values from an age and gender-standardized population of healthy controls. Eighteen steroid metabolites were excreted in significantly higher concentrations in patients with ACC (*n* = 27) compared to patients with ACA (*n* = 107) or other adrenal conditions (*n* = 18). Tetrahydro-11-deoxycortisol (THS) at a cut-off value of 2.35 μmol/24 h differentiated ACC from other adrenal disorders with 100 % sensitivity and 99 % specificity. Elevated urinary excretion of THS was associated with a very high sensitivity and specificity to differentiate between an ACC and a benign adrenal mass. Urinary steroid profiling might be a useful diagnostic test for the evaluation of patients with an adrenal incidentaloma.

## Introduction

An adrenal incidentaloma is an adrenal mass found coincidentally during a radiologic examination performed for reasons other than evaluation for adrenal disease [1]. The estimated prevalence of adrenal incidentalomas ranges from about 0.1 % for general health screening with ultrasonography to 4.4 % in older subjects examined with high-resolution CT scanning [2]. Optimal clinical management of adrenal incidentalomas has not been established as none of the proposed diagnostic algorithms has been validated prospectively [1, 3–5]. Key objectives of these algorithms are to determine whether hormonal overproduction and/or malignant disease is present. The reported frequency of adrenocortical carcinoma (ACC) among patients with adrenal incidentaloma varies from 1.2–12 % [5]. Assessment of malignancy risk is predominantly based on radiological characteristics such as size, shape, and attenuation value of the adrenal mass. In addition, follow-up by monitoring growth rate with repeat CT or MRI-scans at various intervals during 1 to 2 years after discovery is generally advised for those patients not undergoing surgery after the initial evaluation [1, 4–7]. However, a strategy of repeat imaging is associated with certain risks for the patient and is unlikely to be cost-effective [8]. Urinary steroid profiling (USP) might offer an alternative diagnostic tool for discriminating between ACC and non-ACC in individuals presenting with an adrenal incidentaloma, as it has been shown that ACC is often accompanied by alterations in the urinary steroid metabolome [9–11]. Currently, only few studies have examined the potential diagnostic value of USP in individuals harboring an adrenal tumor [12–15]. Except for the study by Arlt et al. [13], these studies described a small number of subjects. Therefore, the primary objective of our study was to determine the diagnostic performance of USP in differentiating ACC from non-ACC in a large cohort of patients with adrenal tumors.

## Patients and Methods

Both Máxima Medical Center (MMC) and University Medical Center Groningen (UMCG) are referral centers for patients with adrenal disease. We retrospectively analyzed all urinary steroid profiles ordered for evaluation of an adrenal tumor at UMCG and MMC from January 1, 2000 until November 1, 2011. In addition, we included all adult patients with ACC diagnosed from January 1, 2011 until January 1, 2014 in whom a baseline urinary steroid profile was performed. The corresponding medical records were examined and the following data were retrieved: age, gender, symptoms, and signs, laboratory measurements (i.e., hormonal tests), imaging studies, treatment, pathology reports, clinical outcome, and follow-up.

Endocrine activity of the tumor was determined based on the results of locally applied hormonal assays. Glucocorticoid excess was defined as an abnormal result of the 1 mg overnight dexamethasone suppression test (cut-off value serum cortisol at 50 nmol/L) and/or as an elevated 24 h urinary free cortisol excretion. Serum aldosterone to plasma renin ratio was determined to screen for primary aldosteronism, serum dehydro-epiandrosteronesulfate (DHEAS), and testosterone were measured to demonstrate hyperandrogenism (cut-off values based on individual age and gender-related upper reference limits). Plasma-free metanephrines or urinary fractionated metanephrines were measured to detect a pheochromocytoma. Tumor size and unenhanced CT attenuation values were collected from the original radiological examination report. In case these were not reported, the original CT studies were re-examined by an experienced radiologist.

The final diagnosis was based on either the pathological examination of the resected adrenal gland or on the clinical course including the results of follow-up imaging studies in patients who did not undergo surgery. In patients with ACC, the Weiss score (a set of nine histopathological criteria with prognostic value in adrenocortical tumors) was extracted from the original pathology reports and disease progression was staged according to the European Network for Study of Adrenal Tumors (ENSAT) staging system [16, 17]. Patients in whom a final diagnosis of pheochromocytoma was made were excluded from the present study.

Gas chromatography/mass-spectrometry (GC/MS) for determination of the USP was performed at the department of laboratory medicine of the UMCG. In summary, free and conjugated steroids were extracted from 1-mL urine by liquid-liquid extraction. Enzymatic hydrolyzation, re-extraction, and chemical derivatization formed methyloxime-trimethyl-silyl ethers from steroid conjugates. An Agilent 5973 instrument operating in selected ion-monitoring (SIM) mode was used to achieve sensitive and specific detection and quantification of 22 selected steroid metabolites [18].

Reference values for USP were established in a group of healthy volunteers (*n* = 240) recruited from the LifeLines cohort, a three-generation population-based study [19]. These subjects were stratified according to gender and age, with age ranging from 20 to 79 years and each decade comprising 40 subjects (male-to-female ratio 1:1).

### Statistical Analysis

Demographical characteristics were assessed using ANOVA for continuous variables and Pearson’s chi-squared test for categorical variables. Between-group differences in steroid excretion were evaluated using the Kruskal-Wallis test followed by post hoc analysis using the Mann-Whitney test with Bonferroni adjustment (*α* = 0.008). Receiver operating characteristics (ROC) curves were generated for those individual urinary metabolites which displayed a significant between-group difference. Sensitivity and specificity were calculated at cut-off values providing highest sensitivity. Cut-off values were compared with age- and gender-dependent reference values. Correlation between histological characteristics and steroid excretion in patients with ACC was calculated using Pearson’s correlation analysis and expressed as Pearson’s *r*. A two-sided *P* value <0.05 was considered to be significant. Data management and statistical analyses were performed using Prism 6.0 (GraphPad Software, La Jolla, USA) and SPSS 19.0 (IBM, Armonk, USA).

## Results

### Patients

We evaluated 152 patients with an adrenal tumor (52 males, 100 females), and demographical characteristics are summarized in Table 1. The following diagnoses were established: adrenocortical carcinoma (ACC; *n* = 27) and adrenocortical adenoma (ACA; *n* = 107). In addition, there was a mixed group of 18 patients with a variety of diagnoses: benign myelolipoma (*n* = 4), metastasis from an extra-adrenal primary tumor (*n* = 4), adrenal hyperplasia (*n* = 2), multinodular hyperplasia (*n* = 2), cavernous lymphangioma (*n* = 1), ganglioneuroma (*n* = 1), hemangioma (*n* = 1), cyst (*n* = 1), non-Hodgkin lymphoma (*n* = 1) and leiomyosarcoma (*n* = 1). The majority of these patients were analyzed because of an adrenal incidentaloma (*n* = 109, 72 %).

**Table 1:** Clinical and radiological characteristics of 152 patients who were evaluated for an adrenal tumor

		Total (*n* = 152)	ACC (*n* = 27)	ACA_functioning_ (*n* = 22)	ACA_non-functioning_ (*n* = 85)	Other (*n* = 18)	*P* value
Age (years) [mean ± SD]		56 ± 13	57 ± 14	50 ± 12	58 ± 12	54 ± 17	0.043*
Sex [*n* (%)]	Male	52 (34)	8 (30)	6 (27)	28 (33)	10 (56)	0.219
	Female	100 (66)	19 (70)	16 (73)	57 (67)	8 (44)	
BMI (kg/m^2^) [mean ± SD]		28.0 ± 5.6	26.7 ± 4.9	29.1 ± 4.4	28.0 ± 5.7	28.8 ± 7.1	0.552
Hormonal overproduction [*n* (%)]	Glucocorticoids	38 (25)	18 (67)	19 (86)	0	1 (6)	–
	Androgens	14 (9)	14 (52)	0	0	0	
	Estrogens	1 (1)	1 (4)	0	0	0	
	Mineralocorticoids	4 (3)	0	3 (13)	0	1 (6)	
Tumor size (cm) [median (range)]		3.5 (0.8–17.0)	10.0 (5.3–17.0)	3.0 (0.9–5.0)	2.8 (0.8–10.0)	7.4 (2.3–14.0)	<0.001**
CT densitometry (HU)[*n*]	≤10	37	0	6	28	3	–
	>10	41	13	2	22	4	
	n/a	74	14	14	35	11	

*P* value for comparison between groups, n/a: unenhanced CT scan was not performed

*ACC* adrenocortical carcinoma, *ACA* adrenocortical adenoma, *BMI* body mass index

*Mann-Whitney *U* test with Bonferroni correction for comparison between individual groups (*α* = 0.008) between ACA_functioning_ and ACA_non-functioning_, *P* = 0.005; other comparisons did not show significant results

**Mann-Whitney *U* test with Bonferroni correction for comparison between individual groups (*α* = 0.008) between ACC and ACA_functioning_, *P* < 0.001; between ACC and ACA_non-functioning_, *P* < 0.001; between ACC and other, *P* = 0.003; between ACA_non-functioning_ and other, *P* < 0.001; and between ACA_functioning_ and other, *P* = 0.001

A diagnosis of ACC was histologically confirmed in 25 subjects (93 %). Median Weiss-score was 6 (range 4-8). In the remaining 2 patients, a clinical diagnosis of ACC was made based on imaging studies showing an adrenal tumor with metastases in combination with biochemical evidence of steroid hypersecretion. At presentation, the tumor stages according to the ENSAT classification were II (*n* = 10), III (*n* = 6) and IV (*n* = 11) [16]. Hormonal overproduction was present in 20 patients (74 %); hypercortisolism (*n* = 18), hyperandrogenism (*n* = 14), and hyperestrogenism (*n* = 1). Histological confirmation was obtained in 28 subjects with ACA (26 %) and in 13 patients from the mixed group (68 %). In the remainder of subjects, a diagnosis of ACA was established by biochemical and imaging studies as well as by clinical follow-up (median 52 months; range 3–167 months). Patients with functioning ACA demonstrated hypercortisolism (*n* = 19) or primary aldosteronism (*n* = 3).

### Radiological Characteristics

Patients underwent radiological imaging with CT (*n* = 126) and/or MRI (*n* = 29). Median tumor size in patients with ACC was 10.0 cm (range 5.3–17.0 cm), significantly larger than the lesion size in patients with ACA (2.9 cm; range 0.8–10.0 cm) or in patients from the mixed group (7.4 cm; range 2.3–14.0 cm, *P* < 0.001 for both; Table 1).

An unenhanced abdominal CT scan was performed in 78 patients (51 %). All adrenal lesions with attenuation value ≤10 HU (*n* = 37) were found to be benign. The 41 adrenal tumors with an unenhanced attenuation value >10 HU included ACC (*n* = 13), ACA (*n* = 24), metastatic lesions (*n* = 2), benign myelolipoma (*n* = 1), and a leiomyosarcoma (*n* = 1). At a cut-off value of 10 HU, unenhanced CT densitometry was 100 % sensitive and 57 % specific for the presence of ACC.

### Steroid Metabolite Excretion

**Fig. 1:**
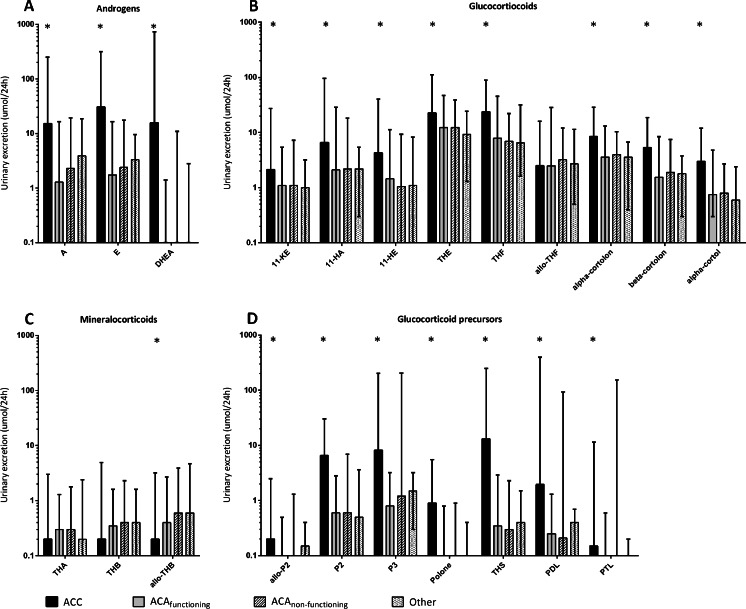
**a**–**d**. Median urinary excretion of 22 metabolites in patients with ACC, functioning adenoma, non-functioning adenoma, or other adrenal tumors organized per steroid class. *A* androsterone, *E* etiocholanolone, *DHEA* dehydroepiandrosterone, *11*-*KE* 11-keto-etiocholanolone, *11*-*HA* 11-hydroxy-androsterone, *11*-*HE* 11-hydroxy-eticholanolone, *THE* tetrahydrocortisone, *THF* tetrahydrocortisol, *allo*-*THF* allo-tetrahydrocortisol, *THA* tetrahydro-11-dehydrocorticosterone, *THB* tetrahydrocorticosterone, *allo*-*THB* allo-tetrahydrocorticosterone, *allo*-*P2* allo-pregnanediol, *P2* pregnanediol, *P3* pregnanetriol, *Polone* epi-pregnanolone, *THS* tetrahydro-11-deoxycortisol, *PDL* pregnanediolone, *PTL* pregnanetriolone. Whiskers indicate range, * indicates significant difference between ACC and other groups

Eighteen steroid metabolites were excreted in significantly larger quantities by patients with ACC compared to patients with non-ACC-related adrenal masses (Fig. 1). In contrast, patients with ACC excreted less of the metabolite allo-tetrahydrocorticosterone (allo-THB) compared to subjects without ACC (*P* = 0.019). There were no significant differences in individual metabolite excretion between the groups with functioning ACA, non-functioning ACA, and the mixed group.

**Fig. 2:**
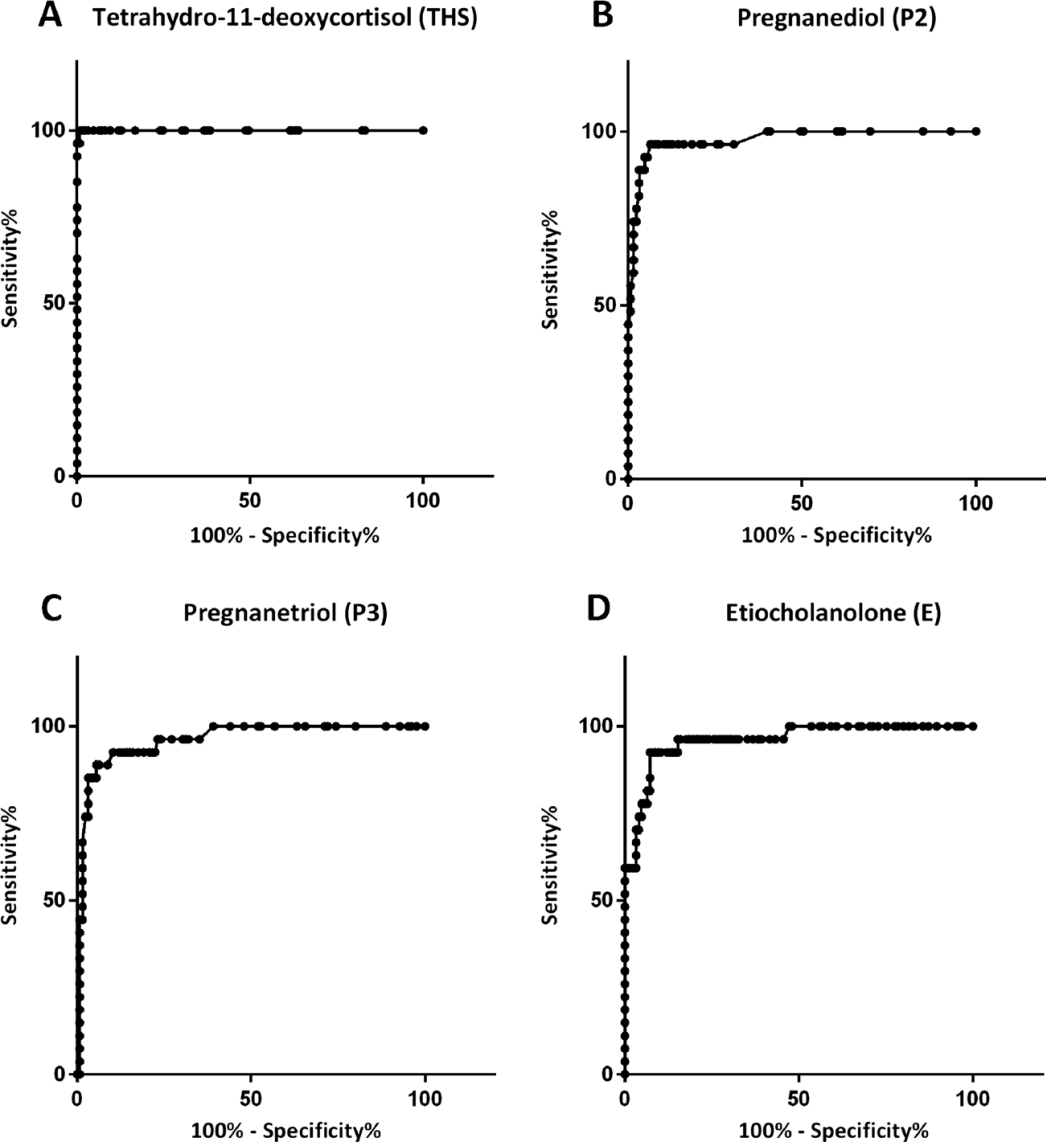
**a**–**d**. Receiver operating characteristic (ROC) curve for tetrahydro-11-deoxycortisol, pregnanediol, pregnanetriol, and etiocholanolone for the diagnosis of adrenocortical carcinoma

**Fig. 3:**
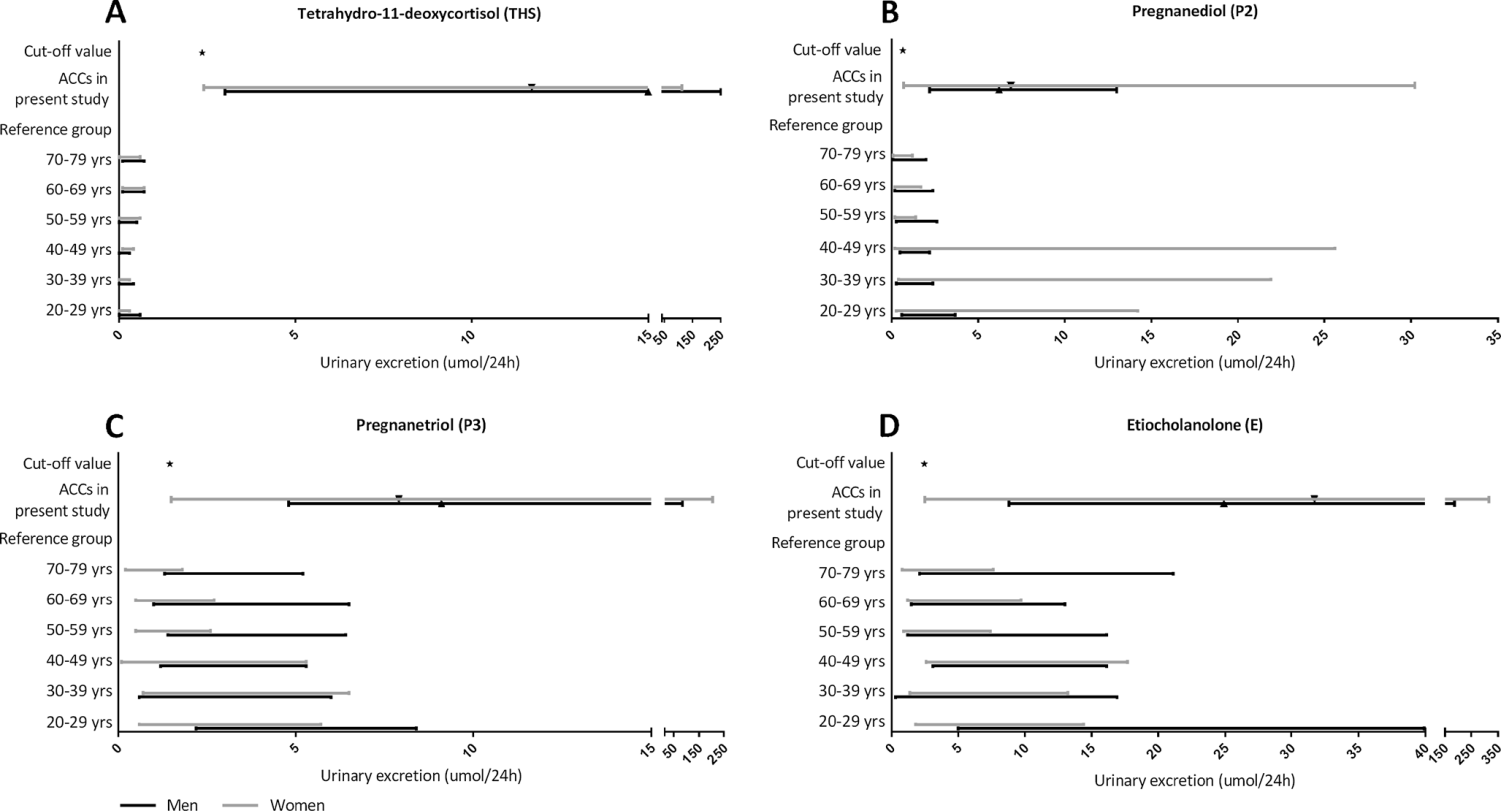
**a**–**d**. Relationship between cut-off values, reference values, and measured urinary excretion of tetrahydro-11-deoxycortisol, pregnanediol, pregnanetriol, and etiocholanolone in 27 ACC patients

ROC-analysis demonstrated that 15 steroid metabolites had a sensitivity >90 % for detecting ACC, of which 7 had a sensitivity of 100 % (i.e., tetrahydro-11-deoxycortisol (THS), pregnanediol (P2), pregnanetriol (P3), etiocholanolone (E), androsterone, tetrahydrocortisol, and tetrahydrocortisone; Table 2). The ROC curve for THS was found to have the largest AUC (1.0), with a sensitivity of 100 % and a specificity of 99 % at a cut-off value of 2.35 μmol/24 h (Fig. 2). This cut-off value significantly exceeded each of the age and gender-specific upper reference limits. Median THS excretion in patients with ACC, ACA, or those belonging to the mixed group was 13.10 μmol/24 h (interquartile range 6.20–35.90 μmol/24 h), 0.30 μmol/24 h (IQR 0.20–0.65 μmol/24 h), and 0.40 μmol/24 h (IQR 0.20–0.63 μmol/24 h), respectively. Excluding patients in whom the adrenal tumor had a CT attenuation value <10 HU (*n* = 37) did not affect the diagnostic performance of THS excretion (AUC 1.0). Figure 3 displays the relationship between cut-off values, reference intervals, and median excretion of four steroid metabolites showing the highest sensitivity and specificity (THS, P2, P3, E).

**Table 2:** Receiver operating characteristics for individual steroid metabolites with sensitivity for detecting ACC >90 %

Metabolite	AUC	Cut-off value (μmol/24 h)	Sensitivity (%)	Specificity (%)
THS	1.000	2.35	100	99
P2	0.975	0.66	100	60
P3	0.960	1.45	100	61
E	0.960	2.47	100	53
A	0.839	0.35	100	14
THF	0.806	2.10	100	5
THE	0.700	3.20	100	4
PDL	0.884	0.15	96	26
11-KE	0.733	0.49	96	18
β-cortolone	0.793	0.45	96	7
α-cortolone	0.760	0.75	96	5
α-cortol	0.789	0.15	96	4
11-HA	0.699	0.15	96	2
11-HE	0.761	0.49	93	25
Polone	0.922	0.15	91	83

*AUC* area under curve, *THS* tetrahydro-11-deoxycortisol, *P2* pregnanediol, *P3* pregnanetriol, *E* etiocholanolone, *A* androsterone, *THF* tetrahydrocortisol, *THE* tetrahydrocortisone, *PDL* pregnanediolon, *11*-*KE* 11-keto-etiocholanolone, *11*-*HA* 11-hydroxy-androsterone, *11*-*HE* 11-hydroxy-eticholanolone, *Polone* epi-pregnanolone

Median THS excretion in patients with ACC and ENSAT stages II, III, or IV was 8.70 μmol/24 h (range 2.40–45.20 μmol/24 h), 10.00 μmol/24 h (range 3.30–39.20 μmol/24 h), and 30.40 μmol/24 h (range 6.20–250.00 μmol/24 h), respectively (*P* = 0.025). Histopathological description of diameter and weight of the ACC was available for 23 and 13 patients, respectively. We found a significant correlation between THS excretion and ACC diameter (*r* = 0.477, *P* = 0.021) and a near-significant correlation between THS excretion and ACC weight (*r* = 0.553, *P* = 0.050).

## Discussion

In a relatively large cohort of patients with primary adrenal tumors, we found that urinary steroid profiling (USP) by gas chromatography/mass-spectrometry discriminates ACC from non-ACC with high diagnostic accuracy. In particular, we demonstrated that measurement of THS had the highest diagnostic test performance with a sensitivity and specificity of 100 and 99 %, respectively. In addition, THS excretion was significantly correlated with ACC tumor size and stage.

It has been demonstrated in several studies that USP may often reveal an increased excretion of steroid metabolites in patients with ACC, even in those without clinical signs of hormonal overproduction [10, 12, 14, 15, 20]. These observations suggest that USP might be a useful diagnostic tool in order to determine whether an adrenal tumor is either malignant or benign. Nearly all studies included a small number of patients with an adrenal tumor, limiting the external validity of these data. So far, only the study by Arlt et al. had a size comparable to the current series [13]. These investigators established a specificity and sensitivity of both 88 % for the nine most differentiating steroid metabolites after conducting a more complex analysis, i.e., generalized matrix learning vector quantization. In addition, they also identified THS as the most discriminative steroid in differentiating benign from malignant adrenal tumors. There are some differences between the present study and the one by Arlt et al. which might explain why we established a higher diagnostic performance for USP. In all our subjects, USP was performed before removal of the primary ACC. In contrast, 22 % of patients with ACC in the study by Arlt et al. were examined after removal of the primary tumor because of the presence of metastases. In view of the here described correlation between tumor size and THS excretion, it seems likely that the difference in diagnostic performance of USP can be partially explained by variation in tumor burden between the two populations at study. Presumably, a gradual increase in urinary THS occurs during development of metastatic disease. During follow-up after primary resection, THS levels below this cut-off value may not exclude the presence of residual or recurrent disease.

The etiology of THS overproduction in ACC remains speculative. Under physiological conditions, ACTH stimulates the conversion of free cholesterol to pregnenolone in mitochondria. Further steroid hormone biosynthesis takes place on the endoplasmic reticulum, except for the final steps in glucocorticoid and mineralocorticoid synthesis [21]. These reactions, catalyzed by CYP11B1 (11-deoxycortisol → cortisol) and CYP11B2 (11-deoxycorticosterone → aldosterone), take place on the inner mitochondrial membrane (IMM) and are stimulated by ACTH [22]. The abundant presence of THS, a metabolite of 11-deoxycortisol, suggests a relative deficiency of otherwise normally functioning CYP11B1 and/or dysfunction of the enzyme itself [13, 23]. Possible explanations for a relative deficiency include increased production of steroid precursors upstream due to malignant proliferation or impaired access of substrate to the IMM. Dysfunction of CYP11B1 may be caused by mutational changes inherent to ACC or diminished ACTH secretion due to negative feedback by increased levels of steroid precursors.

The results of our study suggest that USP could be a valuable diagnostic tool in analyzing an adrenal incidentaloma. Current diagnostic algorithms mainly depend on radiological characteristics for differentiation between benign and malignant adrenal tumors [1, 4–7]. CT scanning, however, has several disadvantages such as exposure to ionizing radiation, adverse effects of radiocontrast (nephropathy, allergic reactions), and costs [8]. Thus, USP might offer a patient friendly, entirely safe, and less expensive alternative diagnostic test for the evaluation of an adrenal incidentaloma. Further validation of this test is warranted and is the main objective of a recently started multicenter prospective study in the Netherlands (NCT02324647).

Like previous studies on this subject, the value of our study is limited by its retrospective design. In addition, selection bias could have influenced the results since it cannot be guaranteed that all patients with an adrenal tumor were evaluated with USP. Moreover, not all diagnoses were confirmed by histopathological examination. This is, however, in agreement with the clinical practice to exclude the diagnosis of ACC in case symptoms or signs suggestive for adrenal malignancy have not occurred during long-term follow-up of the patient.

In conclusion, USP might be a useful diagnostic tool for discriminating between benign and malignant adrenocortical tumors. In particular, increased urinary excretion of THS was associated with an almost perfect diagnostic power. Prospective studies are required to establish the true diagnostic value of USP in patients with an adrenal incidentaloma.
